# Breast composition and dose deposition to fat and fibroglandular tissues are associated with breast side effects after radiation therapy

**DOI:** 10.1016/j.breast.2026.104694

**Published:** 2026-01-07

**Authors:** Tanwiwat Jaikuna, Fiona Wilson, Carmel Anandadas, David Azria, Jenny Chang-Claude, Maria Carmen De Santis, Sara Gutiérrez-Enríquez, Marcel van Herk, Peter Hoskin, Lea Kotzki, Maarten Lambrecht, Zoe Lingard, Petra Seibold, Alejandro Seoane, Elena Sperk, R Paul Symonds, Christopher J. Talbot, Tiziana Rancati, Tim Rattay, Victoria Reyes, Barry S. Rosenstein, Dirk de Ruysscher, Ana Vega, Liv Veldeman, Adam Webb, Catharine M.L. West, Eliana Vasquez Osorio, Marianne C. Aznar

**Affiliations:** a Division of Cancer Sciences, School of Medical Sciences, Faculty of Biology, Medicine and Health, The University of Manchester, Christie NHS Foundation Trust Hospital, Manchester, United Kingdom; b Division of Radiation Oncology, Department of Radiology, Faculty of Medicine Siriraj Hospital, Mahidol University, Bangkok, Thailand; c University Federation of Radiation Oncology of Mediterranean Occitanie, Montpellier Cancer Institute ICM, Université Montpellier, INSERM 1194 IRCM, Montpellier, France; d Division of Cancer Epidemiology, German Cancer Research Center (DKFZ), Heidelberg, Germany; e University Cancer Center Hamburg (UCCH), University Medical Center Hamburg-Eppendorf, Germany; f Radiation Oncology, Fondazione IRCCS Isituto Nazionale dei Tumori, Milan, Italy; g Hereditary Cancer Genetics Group, Vall d’Hebron Institute of Oncology (VHIO), Vall d’Hebron Hospital Campus, Barcelona, Spain; h University Federation of Radiation Oncology of Mediterranean Occitanie, Gard Cancer Institute ICG, CHU Caremeau, Nimes, France; i KU Leuven, Department of Radiation Oncology, Leuven, Belgium; j Medical Physics Department, Vall d’Hebron Hospital Universitari, Vall d’Hebron Barcelona Hospital Campus, Barcelona, Spain; k Department of Radiation Oncology, Mannheim Cancer Center, Medical Faculty Mannheim, University of Heidelberg, Mannheim, Germany; l Leicester Cancer Research Centre, University of Leicester, United Kingdom; m Data Science Unit, Fondazione IRCCS Istituto Nazionale dei Tumori, Milan, Italy; n Radiation Oncology Department, Vall d’Hebron Hospital Universitari, Vall d’Hebron Barcelona Hospital Campus, Barcelona, Spain; o Department of Radiation Oncology, Department of Genetics and Genomic Sciences, Icahn School of Medicine at Mount Sinai, New York, USA; p Maastricht University Medical Center, Department of Radiation Oncology (Maastro Clinic), GROW School for Oncology and Developmental Biology, Maastricht, the Netherlands; q Fundación Pública Galega de Medicina Xenómica, Grupo de Medicina Xenómica (USC), Santiago de Compostela, Spain; r Instituto de Investigación Sanitaria de, Santiago de Compostela, Spain; s Biomedical Network on Rare Diseases (CIBERER), Spain; t Ghent University Hospital, Department of Radiation Oncology, Ghent, Belgium; u Department of Genetics and Genome Biology, University of Leicester, United Kingdom

**Keywords:** Breast composition, Breast density, Breast toxicity, Breast radiotherapy

## Abstract

**Objective:**

Breast comprises different tissues with potentially different dose responses to radiation therapy (RT). This study investigates the correlation between RT dose, breast composition, and side effects from breast RT.

**Material/methods:**

Data from 922 early-stage breast cancer patients who underwent breast-conserving surgery and RT from the REQUITE study were included. Breast pain, oedema, atrophy, and induration were assessed immediately post-RT, one-year, and two-years post-RT. Maximum severity scores for each toxicity were used for analysis. Breast tissue was divided into “*fat*” and “*fibroglandular*” substructures from computed tomography (CT) using a Gaussian Mixture Model. The correlation between breast characteristics, toxicity, dosimetric parameters, and patient and clinical variables was investigated using ordinal regression. The model's fit was evaluated using the Akaike Information Criterion in SPSS v.29.

**Results:**

Breast volume and breast density were associated with increased risk of breast oedema, atrophy, and induration in multivariable analysis (*p<*0.05). Higher mean dose and dose uniformity were observed for fibroglandular compared to fatty tissue at all severity levels, while there was no significant difference in the maximum dose to either substructure. Higher dose deposit to fat was associated with breast pain and oedema, while breast atrophy and induration were associated with dose to fibroglandular tissue. All best-performing toxicity models included dosimetric parameters derived from breast composition.

**Conclusion:**

Breast characterisation offers new insight into the link between dose and toxicity. Breast density and dose parameters from different substructures were associated with different breast toxicity. These findings further support the importance of dose homogeneity of breast RT planning.

## Highlights

• An automated CT-based breast tissue classification was developed using GMM.
• Breast volume and density associated with risk of oedema, atrophy, and induration.
• Breast pain/oedema associated with dose to fat tissue; atrophy/induration associated with dose to fibroglandular tissue.
• Dosimetrics from substructure improved toxicity prediction over whole-breast dose.

## 1 Introduction

Breast cancer treatments, including radiation therapy (RT), reduce local and distant recurrence by about 15 % compared to surgery alone [1]. However, RT can cause adverse effects that compromise quality of life and psychological well-being [2,3]. Skin toxicity, such as dermatitis, is a local effect that arises from damage to epithelial cells in the epidermis by radiation [4,5]. Other toxicities—pain, oedema, atrophy, and induration—are more complex and may involve the subcutaneous layer, including lymphatic and nerve networks [6,7], or other internal tissues. Their manifestation may depend on the radiation dose deposited deep within the breast, and it remains unclear whether the breast composition further complicates this.

The mature female breast comprises adipose tissue (fat), fibroglandular tissue, and a network of nerves, blood, and lymph vessels, each exhibiting distinct radiobiological responses [8,9]. The proportions of breast composition vary depending on age, breast volume, hormones, and menopausal status [10,11]. Larger quantities of fat tissue are normally found in large breast volumes, post-menopausal women, and older people. Moreover, fibroglandular tissue volume is linked to age [12] and can be influenced by hormonal status [13], as observed in a link between breast density and age in the involution process [14]. Therefore, the proportion of breast composition varies for each woman, influencing breast density.

Prior research indicates that spatial dose heterogeneity and hot spots significantly increase radiation-induced skin toxicity [15]. However, few studies have explored how individual breast composition influences side-effect risk. One study suggested that higher adipose content may relate to post-RT breast shrinkage, but no clear correlation was found [16]. We hypothesise that breast composition and substructure dose influence toxicity risk, driven either by different radiobiological responses between fat and fibroglandular tissue or by variations in absorbed dose [11].

In breast cancer screening, density is typically classified using the Breast Imaging-Reporting and Data System (BI-RADS) guidelines into four categories: entirely fatty, containing scattered areas of fibroglandular density, heterogeneously dense, and extremely dense [17]. Clustering patients into these subgroups helps clinicians inform patients of cancer risk or the need for alternative imaging, as dense breasts carry up to 6 times higher risk compared to low-density breasts [[18], [19], [20], [21]]. This system standardises density reporting and its implications in both research and clinical practice. However, automatic assessment of breast density on CT requires prior tissue delineation. Manual delineation is resource-intensive and cumbersome. To investigate links between breast composition and post-RT toxicity, automated BI-RADS classification offers a scalable solution for large patient cohorts.

Previous studies have employed various clustering methods using image processing techniques to auto-segment breast tissue into different composition categories. For instance, fuzzy C-means clustering, k-means clustering, and squeeze-and-excitation networks (based on a convolutional neural network) have been applied to mammographic, magnetic resonance images, and computed tomography (CT) images to classify breast density, providing automated and reliable methods for evaluating breast composition [[22], [23], [24], [25], [26]]. However, most research considered the classification in healthy populations, not in postoperative breast tumours.

This study aims to bridge the gap in current knowledge by proposing a new method for clustering a large CT dataset based on BI-RADS-like categories, in order to explore the link between breast composition, dose deposition in different breast tissue, and risk of breast toxicity post-RT.

## 2 Materials and methods

### 2.1 Patient characteristics

Data from 922 early-stage breast cancer patients who underwent breast-conserving surgery and received RT in the supine position between 2014 and 2017 were used. These data are part of the prospective multi-centre study REQUITE (www.requite.eu), which included 18 treatment centres from Europe and the United States. REQUITE was registered at www.controlled-trials.com (ISRCTN98496463) and was approved by local ethics committees in participating countries (for the UK: NRES Approval 14/NW/0035) [27].

Due to inconsistent or missing breast contours across centres, ipsilateral and contralateral breast volumes were retrospectively generated via atlas-based auto-segmentation using a 20-patient template [28]. Following ESTRO guidelines [29], each breast contour was cropped 5 mm from skin to ensure consistency for subsequent analysis.

Breast pain, oedema, atrophy, and induration were prospectively assessed immediately after completing RT, at one-year and two-year post-RT. Clinicians evaluated the severity of breast toxicity following the Common Terminology Criteria for Adverse Events (CTCAE) v4.0 [30]. Patients completed the BR23 questionnaire for the quality of life in breast cancer patients developed by the European Organisation for Research and Treatment of Cancer (EORTC) [31]. The severity was recorded on a four-point scale, with *no toxicity* corresponding to grade 0 (CTCAE)/“not at all” (BR23), *mild* to grade 1/“a little”, *moderate* to grade 2/“quite a bit”, and *severe* to grade 3/“very much”. The maximum score between clinician and patient's records was selected for our analysis to maximise the severity event. Patient's characteristics and toxicity rate in this study are shown in Supplementary Table A1-2.

### 2.2 Global Breast Classification

**Fig. 1:**
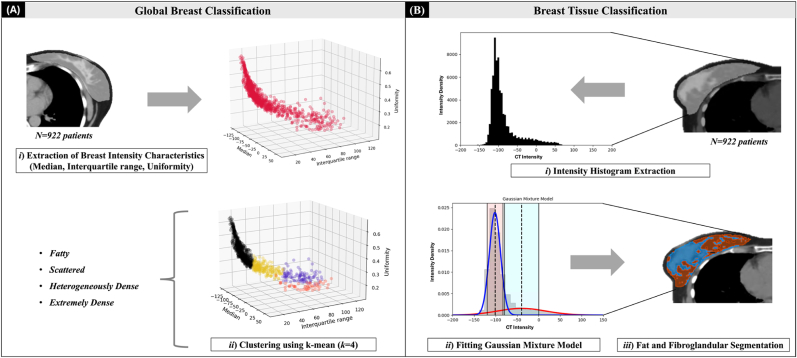
Proposed method to investigate breast composition. (A) Global Breast Classification: breasts were categorised into four clusters following BI-RADs categories by (*i*) extracting breast intensity characteristics from individual breast contour and (*ii*) clustering breast intensity characteristics of each patient into four clusters. (B) Breast Tissue Classification: breast composition was classified for each patient by (*i*) extracting breast intensity histogram from the breast contour, then (ii) fitting the Gaussian Mixture Model with two components to consider the probability of upper and lower threshold of fat and fibroglandular intensity, and (*iii*) applying the binary thresholding to segment fat and fibroglandular tissue using patient-specific intensity threshold.

We categorised patient breasts into four clusters inspired by BI-RADS: fatty, scattered, heterogeneously dense, and extremely dense. Within each breast contour, intensity parameters (median, IQR, and uniformity) were extracted using PyRadiomics v3.1.0 [32]. K-means clustering (*k=4*) grouped the cohort into four clusters by design (Fig. 1A). To validate clustering and investigate intensity characteristics, breast tissue was also classified as fat or fibroglandular tissue (see following section). Analyses were performed in ipsilateral breast and repeated in contralateral breast, as surgery and clips could affect ipsilateral characterisation. We assessed whether significant differences existed between ipsilateral and contralateral breast characteristics, and clustering outputs for patients with both breasts were present in the planning CT.

### 2.3 Breast tissue classification

On average, over 97 % of the adult female breast is composed of fat and fibroglandular tissues [33], which differ in density and therefore in CT intensity. To classify each voxel within breast contour, we developed the intensity histogram–based method (Fig. 1B). Expecting two peaks—fat (lower-intensity) and fibroglandular tissue (higher-intensity)—we applied a Gaussian Mixture Model (GMM) with two components, fitted individually for each patient. Patient-specific intensity ranges were defined using two standard deviations (SD) from each component mean. When overlap occurred, voxels were prioritised as fat tissue, since fat peak was narrower and more distinct than fibroglandular peak. The volume of each substructure was reported as a percentage to the complete breast volume. Differences in breast composition across clusters were evaluated using one-way ANOVA with Tukey's post hoc analysis, and ipsilateral versus contralateral differences were examined using the Wilcoxon test.

### 2.4 Dose deposition to breast substructures

Planning RT dose DICOM files were collected from multiple centres as part of the REQUITE breast cohort. Planning doses were calculated using different dose calculation algorithms, dose grids, and treatment planning systems for photon and electron, depending on the availability of TPS at each centre. The physical dose was converted to a biological equivalent dose in 2 Gy fractions (EQD_2_) to account for differences in fractionation using the Linear-Quadratic Model with α/β=1.7Gy [34]. We followed a similar approach to the previous section by quantifying the mean, maximum, and uniformity of both physical dose and EQD_2_ deposition for each substructure and patient. The uniformity of the dose is the sum of the squares of the dose in each voxel (*i*) within the region of interest, with probability p(*i*) in the total number of doses *n,* and this uniformity represents the homogeneity of the dose distribution, shown in equation (1) [32]. These dose metrics, derived from substructures, were compared with the complete breast contour at each toxicity severity.

(1) $$uniformity=\sum_{i=1}^{n}{p\left(i\right)}^{2}$$

### 2.5 Association of breast composition and breast toxicity

In this analysis, we considered four outcomes—breast pain, oedema, atrophy, and induration—at three time points: immediately after RT, and at one- and two-years post-RT. The first timepoint captured acute toxicity, while the latter addressed late effects. To determine associations with breast composition and dose, we conducted the following steps. First, univariable ordinal logistic regression was performed for each outcome against breast density, fat and fibroglandular tissue volumes, and mean, maximum, and uniformity of dose, evaluated for both substructures and the whole breast. Second, we identified the optimal variable set to complement demographic and treatment factors. A baseline model with demographic and treatment variables was constructed, followed by a final model incorporating selected breast composition and dosimetric variables, as listed above, through forward stepwise selection using Akaike Information Criteria (AIC). Variables were first screened for multicollinearity, retaining those with correlations below 0.9 (Supplementary Table C3). We then applied forward-stepwise selection to dosimetric variables while fixing demographic and clinical data from the baseline model. Model discrimination was compared using AIC. Backwards-stepwise method was reproduced to strengthening confidence in the results (Supplementary Table C4). Analyses were conducted in IBM SPSS v.29, with significance set at *p<0.05*. It is important to note that the aim of this study was not to develop a predictive model per se, but to examine the independent value of breast composition as a parameter.

## 3 Results

### 3.1 Global breast and breast tissue classifications

**Fig. 2:**
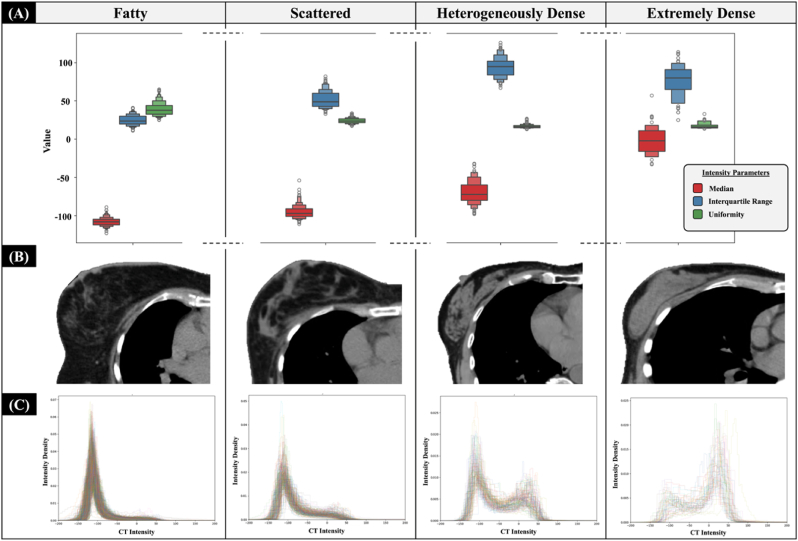
Global breast classification based on BI-RADS-like classes. (A) Distribution of the breast characteristics, including median, interquartile range, and uniformity of CT intensity. (B) An example of a CT image of a representative in each cluster, and (C) CT intensity histogram profiling of the intensity distribution within the contours. Note that (A–C) analysis was performed using the ipsilateral breast contours.

**Fig. 3:**
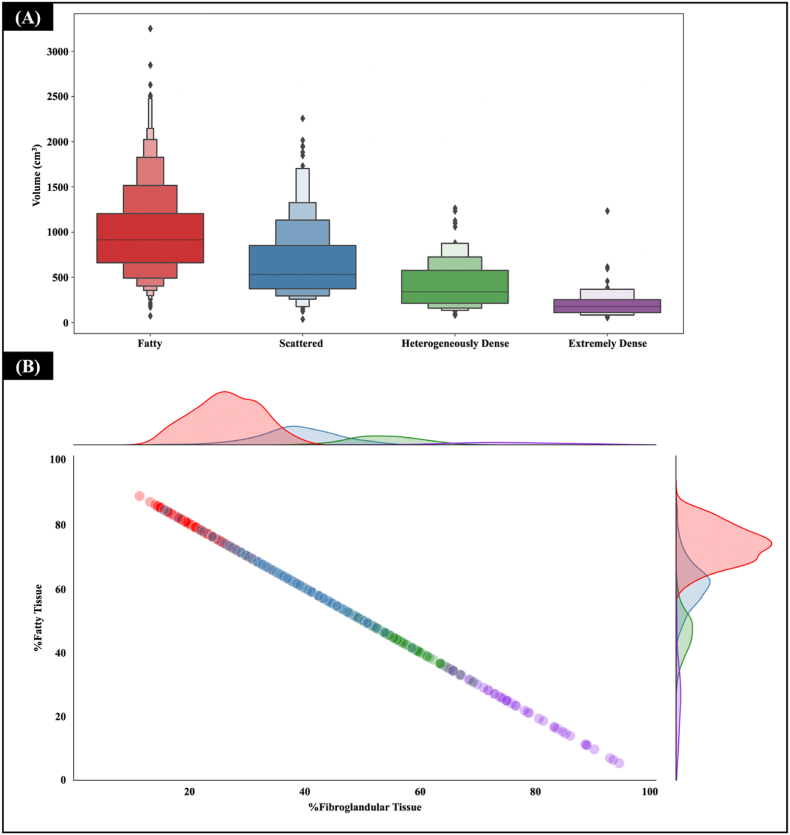
The characteristics of the ipsilateral breast in four breast density clusters: (A) the volume of the complete ipsilateral breast contour and (B) the percentage of fat and fibroglandular tissues of each breast density cluster.

K-means clustering using median, IQR, and intensity uniformity identified four BI-RADS-like breast clusters (Fig. 2), demonstrating the method's feasibility. The lowest median intensity (−108 ± 5 HU), narrowest IQR (25 ± 7 HU), and highest uniformity (40 ± 1 %) were found in fatty cluster. As fibroglandular proportion increased, median intensity increased, peaking in the extremely dense cluster, while uniformity declined. The heterogeneously dense cluster displayed the widest IQR and lowest uniformity, consistent with expectations. Breast volume was greatest in the fatty cluster and decreased continuously with increasing density (Fig. 3A). A linear relationship was observed between fat and fibroglandular tissue proportions: fatty clusters had the highest fat and lowest fibroglandular percentage, while extremely dense clusters showed the reversed pattern (Fig. 3B).

**Fig. 4:**
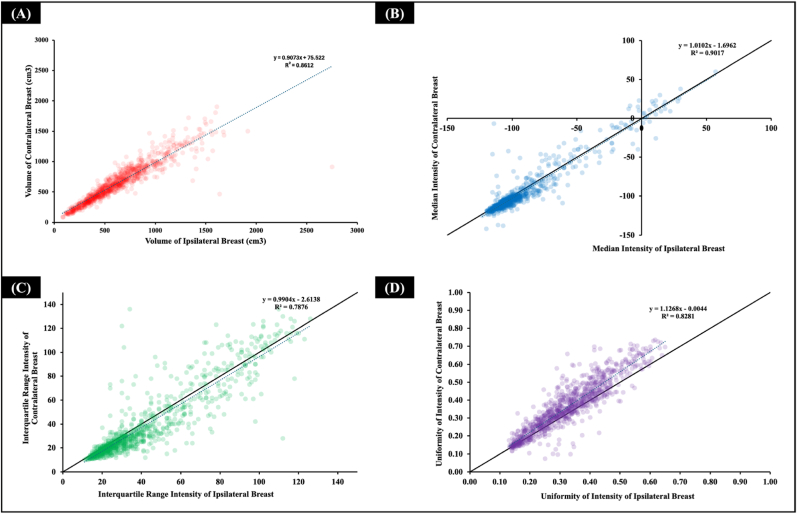
Comparisons between the ipsilateral and contralateral breast. (A) volume, (B) median intensity, (C) interquartile range intensity, (D) uniformity. *Note: the contralateral breast was not fully covered by the field of view of the CT image in 12 patients.*

Contralateral breast contours could be generated in 910/922 patients, as shown in Fig. 4. Ipsilateral breasts were slightly smaller than the contralateral ones, with their volume being on average 90.7 % the volume of the contralateral breasts (*R*^*2*^ = 0.86). Despite this difference, the median intensities were very similar, on average within 1 % (*R*^*2*^ = 0.90). The interquartile intensity presented larger variations, with small average differences (within 1 %) but with a slightly weaker correlation (*R*^*2*^ = 0.79). Uniformity of intensities presented a shift, with contralateral breasts on average 12 % more uniform than ipsilateral breasts (*R*^*2*^ = 0.82), as expected after breast surgery. Regarding substructures, the CT intensity of the ipsilateral breast was lower than the contralateral breast, ∼2 (IQR 4) in fat and 3 (IQR 17) in fibroglandular tissue, Table 1. The individual intensity histogram of each patient is shown in Supplementary Figure B1. The confusion matrix analysis indicates that the scattered and heterogeneously dense clusters were less accurately reproduced when using contra- and ipsilateral breast contours for categorisation, whereas fatty and extremely dense clusters exhibit a high reproducibility (Supplementary Table B1).

**Table 1:** Mean and standard deviation of CT intensity of fat and fibroglandular tissues in the ipsilateral and contralateral breast.

Cluster	Ipsilateral Breast mean (SD)		Contralateral Breast mean (SD)	
	Fat tissue	Fibroglandular tissue	Fat tissue	Fibroglandular tissue
**Fatty**	−110 (5)	−52 (12)	−112 (8)	−54 (24)
**Scattered**	−105 (7)	−33 (10)	−107 (7)	−29 (50)
**Heterogeneously Dense**	−101 (7)	−13 (11)	−101 (9)	−2 (35)
**Extremely Dense**	−76 (25)	9 (15)	−69 (24)	22 (28)

### 3.2 Association of breast composition, dose deposition, and breast toxicity

Univariable analysis revealed that median breast density from complete breast contour and substructure dose were significantly associated with breast toxicities (Supplementary Table C.1). Differences in volume and dosimetric parameters between complete breast and substructures were observed across toxicity levels, especially in the high-fat cluster and less in extremely dense clusters (Supplementary Figures C.1–2). The volume and dosimetric parameters of breast, fat tissue, and fibroglandular tissue increased with greater toxicity severity in most BI-RADS-like clusters, except for the extremely dense cluster. Toxicity severity increased with larger breast volumes, higher mean breast dose, and less dose uniformity. In extremely dense clusters, fibroglandular volume significantly decreased with increasing induration severity.

**Fig. 5:**
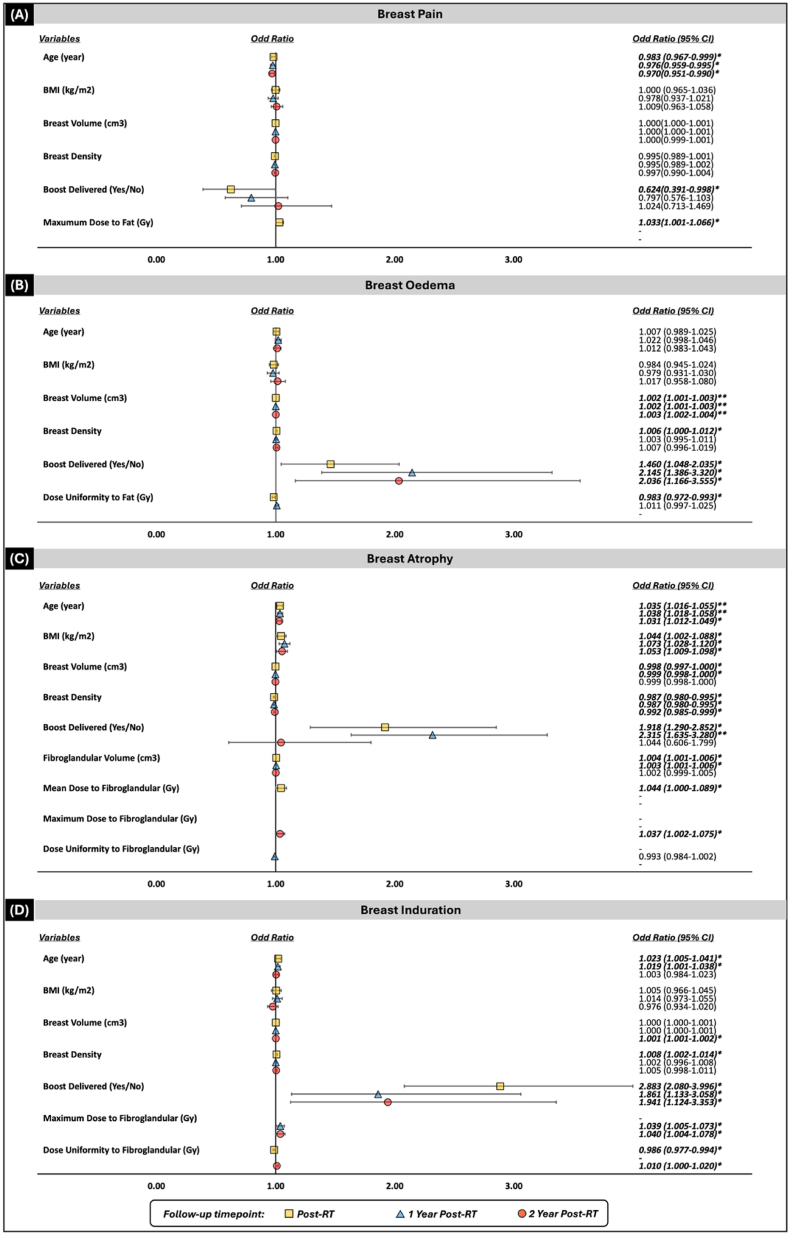
The best-performing models from multivariable ordinal logistic regression showing the association between studied variables and breast toxicity outcomes at each time point for (A) pain, (B) oedema, (C) atrophy, and (D) induration. Note: ∗*p<0.05* and ∗∗*p<0.001*.

The best-performing multivariable model, as shown in Fig. 5, shows that clinical and treatment variables, including age, breast volume, and boost delivered, were significantly associated with breast toxicities. For the complete breast contour analysis, breast density is associated with a higher risk for acute breast oedema, acute breast induration, and acute and late breast atrophy in the best-performing multivariable analysis model. The result of multivariable model when considering in EQD_2_ form was similar to the physical dose, *supplementary material* Figure C3.

Additionally, including breast density and dose deposits in substructure tissues increased the model's robustness. In substructure analysis, larger fibroglandular tissue volume increased the risk of acute and late breast atrophy at one-year post-RT. Higher dose deposits to fat tissue were associated with acute breast pain, and less uniformity of dose deposits to fat tissue was associated with acute breast oedema. Conversely, high-dose deposit in the fibroglandular tissue was associated with breast atrophy and induration. The best model's AIC score decreased from 0.1 to 4.3 points compared with the baseline model (which includes only demographic/treatment variables) and was about 1 point lower than the model including the complete breast dosimetric parameters (Supplementary Table C2).

## 4 Discussion

This study demonstrated that it is feasible to automatically cluster patients in “BI-RADS-like” categories using the k-means method based on breast intensity characteristics (median, interquartile range, and uniformity of breast intensity). Moreover, we described a fast and feasible method to further classify individual breast composition into fat and fibroglandular tissues in a large cohort using GMM without requiring additional training datasets.

K-means clustering was successful in a large multicentre dataset with diverse patient and planning characteristics. However, clusters may vary in new datasets. Categorising patients into BI-RADS is subject to a lack of reproducibility and inter-observer variation. Our analysis primarily included patients of 94 % European ancestry [27]; including a broader population could improve generalisability and applicability across populations.

The outcomes investigated in this study were prospectively collected in the REQUITE study, which provided both clinician-report outcomes (CRO) and patient-report outcomes (PRO). Previous research focused only on PRO [35]. Combining the severity from both reports could increase the evidence of toxicity and provide an excellent opportunity to observe outcomes in low-toxicity datasets. The agreement between CRO and PRO remains controversial, as *exemplified by the disagreement between CRO and PRO in our cohort* (Supplementary Table B2). Some studies have found correlations between the reports, while others have shown disagreement [36,37]. Therefore, using both reports will ensure all toxicity severity is captured and may mitigate the discrepancy.

Our study suggests that lower breast density increases the risk of breast atrophy, while higher density increases risk of oedema and induration post-RT. Additionally, volume and dose to different breast substructures are associated with various breast toxicities post-RT. Specifically, dose to fat tissue is more associated with breast oedema, whereas dose to fibroglandular tissue is linked to atrophy and induration. This study investigated dose uniformity from the total plan dose and the role of the boost in multivariable analysis. The delivery of a boost was not associated with higher breast toxicity rates, e.g., in breast pain. However, the degree of high-dose overlaps with fibroglandular tissue consistently showed a directional relationship with induration.

Regardless of their direction, stepwise variable selection methods are often ineffective when many potential variables exist [38]. However, as our goal was not to create a prediction but to demonstrate the added value of new variables beyond the baseline model, this limitation was less critical. While this approach was appropriate for our study, future research aiming to build predictive models may require more robust methods, such as LASSO or Ridge regression, to identify the best-fitting model for breast toxicity prediction and account for larger variable spaces.

This study demonstrates the potential of combining breast composition substructure parameters to predict breast toxicity risk in individual patients. Our findings align with prior research linking poor cosmesis rate to older age, larger breast volume, and heterogeneous dose distribution [[39], [40], [41], [42], [43], [44], [45], [46]]. However, we found a significant association between decreased breast volume and the risk of breast atrophy post-RT. Thus, while breast volume influences toxicity outcomes, our results also highlight breast density as an independent factor impacting toxicity development—an association not previously identified.

Patients with large breast volumes are mainly in the fatty breast cluster (Fig. 3). With a high proportion of fat tissue in this cluster, it is possible that the radiation could damage and significantly reduce the number of fat cells, which are more sensitive to radiation compared with stromal cells, leading to poorer breast cosmesis [45,46]. In fact, larger breast volumes could potentially produce more dose heterogeneity and the presence of hot spots than smaller breast volumes [47]. Our result is in line with the recognised need to ensure homogeneous dose distribution in breast RT planning to reduce the risk of developing acute and late breast toxicity.

Our research identified a diverse range of fat and fibroglandular compositions among patients of almost homogeneous ethnicity, as the population in this study is of European ancestry [27]. This study developed a unique classification threshold for individual patients based on their breast intensity characteristics, departing from previous studies that used fixed thresholds for each tissue [23,24] and using a qualitative visualisation for clustering breast density patients into clusters [16,35]. However, by enabling individualised thresholds, this pipeline could be investigated in more diverse populations.

Several methods have been proposed to identify fat and fibroglandular tissues in the breast, including connected-component image analysis [48] and Gaussian classification. Nelson et al. applied a two-compartment Gaussian fit for CT breast composition segmentation [49] and used fixed Hounsfield unit (HU) ranges for tissue classification [50]. They calculated population-based *‘optimal*’ CT values for skin, fat, and glandular tissue, while Belardo et al. relied on literature-derived values. However, HU values differ across tissue types; for instance, subcutaneous fat ranges from −140 to −70 HU [51]. We observed that ‘*optimal*’ CT numbers varied between patients. Thus, fixed thresholds or differing CT acquisition protocols in RT may reduce the accuracy and generalisability of breast substructure segmentation.

Breast composition classification depends on contouring and CT quality. Variations in methods, such as including or excluding skin, influence density extraction and artefacts. As observed in published studies, estimates of skin dose are not very reliable in conventional treatment planning systems [52,53], and our study aims to investigate breast toxicity below the skin. Therefore, a 5 mm skin cropping was applied in our study.

Seroma has water-like HU that can overlap with fibroglandular tissue. Because expert manual contouring was not feasible in a large cohort, we did not contour the seroma. As a result, seroma voxels were not explicitly excluded and, when present, may have been assigned to the fibroglandular tissue, potentially biasing the proportion. To limit misclassification, the GMM was fitted within a restricted, per-case HU window centred on the mean HU of fat and fibroglandular tissue with ±2 SD, and HU outliers were ignored. We did not quantify the surgery-affected (“disturbed”) volume. Surgical clips produce a higher HU value and fall outside the fitting window, so they did not enter the composition estimate. Our study also evaluates breast toxicity relative to planning dose distribution, which may be influenced by the choice of dose calculation algorithms, grid size, setup uncertainties, and anatomical changes. Our study did not recalculate all plans using a single calculation algorithm to compensate for this influence, but instead reflected real-world clinical situations. However, dose calculation parameters should be recorded in future large/multi-institute clinical trials to eliminate this uncertainty.

A limited number of studies have investigated the association between breast toxicity and breast density/composition [16,35,54]. Our results align with Kuzmiak CM et al., showing that higher density increases the risk of oedema [54], while another study found no breast composition correlation; only seroma being the potential factor [16]. While previous dosimetric studies have noted small intrabreast differences between adipose and glandular tissues when using dose-to-medium calculations [55] and negligible difference found in adipose tissue when using difference dose calculation algorithm (dose-to-water vs dose-to-medium) [56], to our knowledge, no study has systematically assessed dose deposition according to patient-level breast composition categories or investigated whether such differences translate to variations in toxicity outcomes. Therefore, our research is the first to examine the association between specific parameters of dose deposition in different breast compositions and breast toxicity outcomes.

Breast composition varies among patients, and clustering by density may help predict toxicity risks. This study supports precision RT by guiding clinicians to assess tissue-specific doses and minimise toxicity. Customised RT plans based on breast composition could improve outcomes through personalised strategies tailored to risk profiles. External validation is needed to influence planning and patient communication.

## 5 Conclusion

Automating BI-RADS-like breast cluster and breast tissue classification on RT CT images is achievable. This enables clinicians to assess substructure dose distribution, which influences toxicity outcomes after RT. Incorporating substructure dosimetric parameters enhances prediction models, supporting improved breast RT planning and informing patients about individual toxicity risks.

## CRediT authorship contribution statement

**Tanwiwat Jaikuna:** Writing – review & editing, Writing – original draft, Visualization, Software, Project administration, Methodology, Investigation, Formal analysis, Conceptualization. **Fiona Wilson:** Writing – review & editing, Writing – original draft, Methodology, Conceptualization. **Carmel Anandadas:** Writing – review & editing, Writing – original draft, Methodology, Conceptualization. **David Azria:** Writing – review & editing, Resources, Data curation. **Jenny Chang-Claude:** Writing – review & editing, Resources, Data curation. **Maria Carmen De Santis:** Writing – review & editing, Resources, Data curation. **Sara Gutiérrez-Enríquez:** Writing – review & editing, Resources, Data curation. **Marcel van Herk:** Writing – review & editing, Supervision. **Peter Hoskin:** Writing – review & editing, Supervision. **Lea Kotzki:** Writing – review & editing, Resources, Data curation. **Maarten Lambrecht:** Writing – review & editing, Resources, Data curation. **Zoe Lingard:** Writing – review & editing, Resources, Data curation. **Petra Seibold:** Writing – review & editing, Resources, Data curation. **Alejandro Seoane:** Writing – review & editing, Resources, Data curation. **Elena Sperk:** Writing – review & editing, Resources, Data curation. **R Paul Symonds:** Writing – review & editing, Resources, Data curation. **Christopher J. Talbot:** Writing – review & editing, Resources, Data curation. **Tiziana Rancati:** Writing – review & editing, Resources, Data curation. **Tim Rattay:** Writing – review & editing, Resources, Data curation. **Victoria Reyes:** Writing – review & editing, Resources, Data curation. **Barry S. Rosenstein:** Writing – review & editing, Resources, Data curation. **Dirk de Ruysscher:** Writing – review & editing, Resources, Data curation. **Ana Vega:** Writing – review & editing, Resources, Data curation. **Liv Veldeman:** Writing – review & editing, Resources, Data curation. **Adam Webb:** Writing – review & editing, Resources, Data curation. **Catharine M.L. West:** Writing – review & editing, Supervision, Resources, Data curation. **Eliana Vasquez Osorio:** Writing – review & editing, Supervision, Methodology, Conceptualization. **Marianne C. Aznar:** Writing – review & editing, Supervision, Methodology, Conceptualization.

## Declaration of competing interests

The authors declare that they have no known competing financial interests or personal relationships that could have appeared to influence the work reported in this paper.
