# Supplementary material for: Breast composition and dose deposition to fat and fibroglandular tissues are associated with breast side effects after radiation therapy
Source: Breast. 2026 Jan 7;85:104694. doi: 10.1016/j.breast.2026.104694 (PMC12830329; doi:10.1016/j.breast.2026.104694)
Supplement: Multimedia component 1 [file mmc1.docx]

**Supplementary Materials A**

**Table A.1** Patient, treatment, and clinical demography of investigated 922 breast cancer patients.

| **Demographic** | **Median (IQR) / %** |
| --- | --- |
| **Number of Patient** | 922 |
| **Age (year)** | 59 (16) |
| **Breast Volume (cm^3^)** | 737 (634) |
| **Body Mass Index (Kg/cm^2^)** | 25.4 (6.6) |
| **Menopausal Status**  Pre  Post | 78.8%  21.2% |
| **Staging**  T  N  M | Tis: 16.4%, T1: 63.3%, T2: 16.2%, T3: 0.4%, NA: 3.7%  N0 or NX: 83.4%, N1: 13.6%, N2: 1.7%, N3: 0.4%, NA: 0.9%  M0 or MX: 91.9%, NA: 8.1% |
| **Estrogen Receptor Status** | Positive: 81.2%, Negative: 11.1%, NA: 7.7% |
| **Progesterone Receptor Status** | Positive: 49.8%, Negative: 17.4%, NA: 32.8% |
| **HER-2 Status** | Positive: 8.0%, Negative: 77.3%, NA: 14.7% |
| **Breast Treatment Site**  Left side treated  Right side treated | 52%  48% |
| **Axillar Lymph Node Surgery**  Yes  No | 89%  11% |
| **Radiotherapy**  Planning Technique  Irradiation Technique  Dose Fractionation  Boost status | 3D-CRT: 50.5%, Complex (FiF, IMRT, VMAT): 49.5%  Whole breast (89%), Whole breast with SCF (11%)  Conventional (1.8-2.0Gy): 54.5%,  Hypofractionation (>2Gy): 45.5%  Boost: 50.9% including Simultaneous integrated boost (122), Sequential electron boost (103), Sequential photon boost (105), Sequential photon and electron boost (137), and No Boost: 49.1% |
| **Systemic Treatment**  **Chemotherapy** | 75%  17% |

***Note:*** *3D-CRT: 3-dimensional conformal radiotherapy, FiF: Field in field technique, IMRT: Intensity-modulated radiotherapy, IQR: Inter Quartile Range, VMAT: Volumetric-modulated radiotherapy, SCF: Supraclavicular fossa*

**Table A.2** The rate of maximum severity of breast toxicity derived from the combined reports between clinician and patient-reported outcomes in the investigated cohort of 922 breast cancer patients.

| **Breast Toxicity** | **Severity at post-RT (Number of patients)** | | | | **N** |
| --- | --- | --- | --- | --- | --- |
|  | **0** | **1** | **2** | **3** |  |
| **Pain** | 167 | 530 | 173 | 52 | 922 |
| **Oedema** | 350 | 449 | 97 | 26 |  |
| **Atrophy** | 627 | 206 | 86 | 3 |  |
| **Induration** | 578 | 300 | 40 | 4 |  |
|  | | | | | |
|  | **Severity at one-year post-RT (Number of patients)** | | | |  |
|  | **0** | **1** | **2** | **3** |  |
| **Pain** | 329 | 404 | 93 | 37 | 863 |
| **Oedema** | 581 | 218 | 48 | 16 |  |
| **Atrophy** | 538 | 220 | 67 | 8 | 833 |
| **Induration** | 455 | 310 | 57 | 11 |  |
|  | | | | | |
|  | **Severity at two-year post-RT (Number of patients)** | | | |  |
|  | **0** | **1** | **2** | **3** |  |
| **Pain** | 370 | 298 | 85 | 27 | 780 |
| **Oedema** | 622 | 127 | 26 | 9 | 784 |
| **Atrophy** | 411 | 247 | 92 | 13 | 763 |
| **Induration** | 464 | 241 | 50 | 8 |  |

**Supplementary Material B**


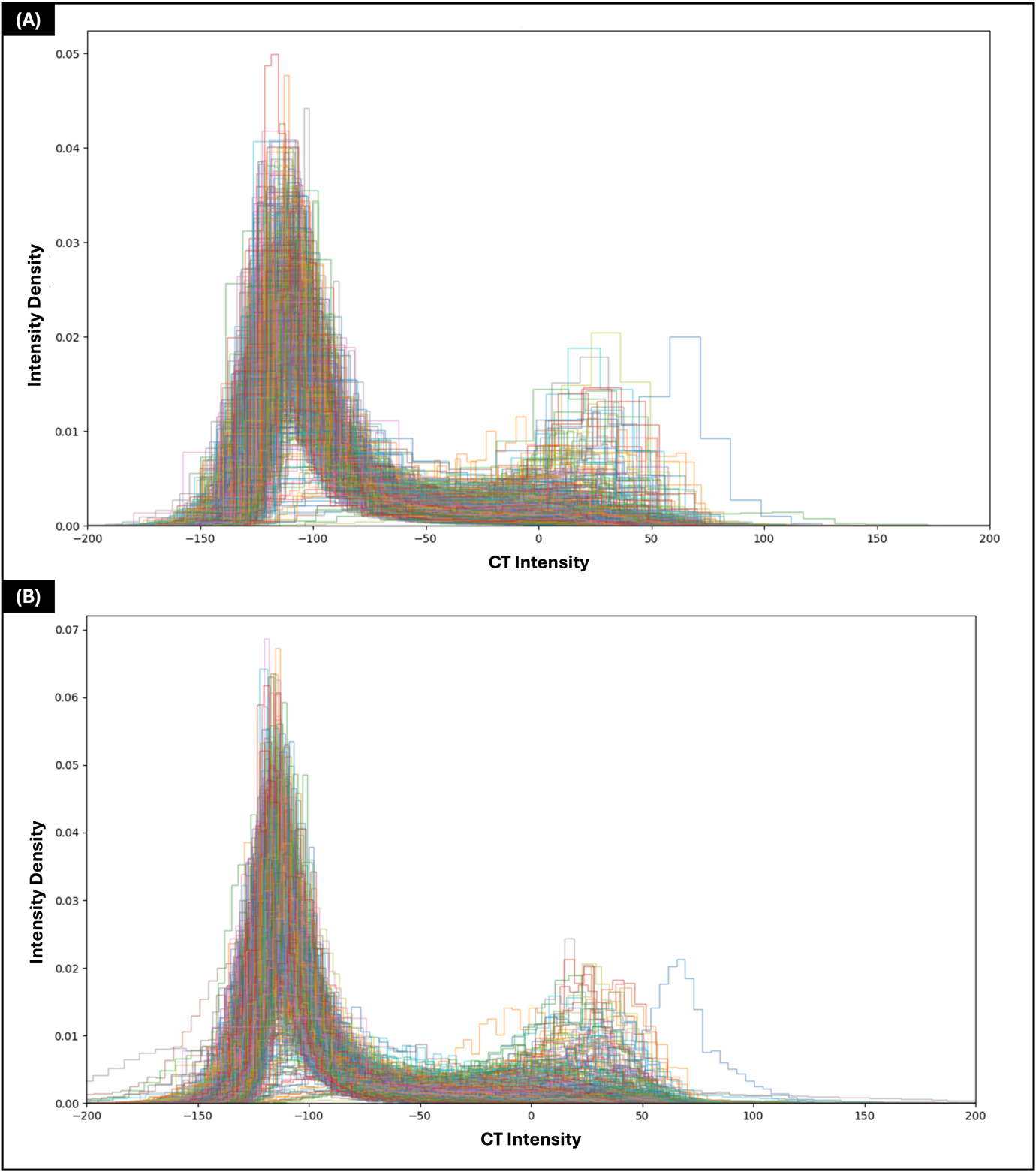


**Figure B.1** The intensity profile of (A) the contralateral breast and (B) the ipsilateral breast.

**Table B.1** Matched cases analysis of clustering breast density between using the contralateral breast and the ipsilateral breast.

| **Contra/Ipsilateral Breast** | **Fatty** | **Scatter** | **Heterogenous** | **Extremely Dense** |
| --- | --- | --- | --- | --- |
| **Fatty** | **553** | 72 | 1 | 0 |
| **Scatter** | 2 | **8** | 68 | 10 |
| **Heterogenous** | 14 | 112 | **27** | 0 |
| **Extremely Dense** | 0 | 0 | 2 | **39** |

*Note: The ideal confusion matrix must have an observed number in bold, while others should be zero.*

**Table B.2** The agreement frequency of severity report between clinicians and patients in (A) breast swelling and (B) breast pain at baseline, immediately post-RT, 1- and 2-year post-RT.

| **(A)** | **Timepoint** | | **Baseline** | | | |  | **PostRT** | | | |  | **1-year post-RT** | | | |  | **2-year post-RT** | | | |
| --- | --- | --- | --- | --- | --- | --- | --- | --- | --- | --- | --- | --- | --- | --- | --- | --- | --- | --- | --- | --- | --- |
| **Breast Swelling** | **Severity** | | **CRO** | | | | | | | | | | | | | | | | | | |
|  |  |  | 0 | 1 | 2 | 3 |  | 0 | 1 | 2 | 3 |  | 0 | 1 | 2 | 3 |  | 0 | 1 | 2 | 3 |
|  | **PRO** | 0 | 545 | 41 | 1 | 0 |  | 554 | 43 | 1 | 0 |  | 511 | 69 | 8 | 0 |  | 568 | 44 | 2 | 0 |
|  |  | 1 | 180 | 38 | 3 | 0 |  | 180 | 38 | 3 | 0 |  | 81 | 53 | 5 | 0 |  | 57 | 19 | 6 | 0 |
|  |  | 2 | 40 | 18 | 2 | 0 |  | 40 | 18 | 2 | 0 |  | 14 | 13 | 7 | 0 |  | 6 | 9 | 4 | 0 |
|  |  | 3 | 14 | 5 | 1 | 0 |  | 14 | 5 | 1 | 0 |  | 5 | 7 | 4 | 0 |  | 4 | 4 | 1 | 0 |
|  |  |  | *NA=34* | | | |  | *NA=23* | | | |  | *NA=145* | | | |  | *NA=190* | | | |
| **(B)** |  | 0 | 293 | 43 | 1 | 1 |  | 62 | 111 | 34 | 14 |  | 129 | 117 | 28 | 12 |  | 164 | 91 | 28 | 7 |
| **Breast Pain** |  | 1 | 0 | 430 | 13 | 5 |  | 77 | 322 | 80 | 20 |  | 109 | 203 | 29 | 13 |  | 89 | 128 | 24 | 8 |
|  |  | 2 | 0 | 0 | 75 | 0 |  | 22 | 77 | 53 | 10 |  | 28 | 25 | 22 | 7 |  | 28 | 20 | 19 | 4 |
|  |  | 3 | 0 | 0 | 0 | 23 |  | 6 | 20 | 6 | 8 |  | 6 | 17 | 0 | 2 |  | 5 | 4 | 2 | 3 |
|  |  |  | *NA=38* | | | |  | *NA=24* | | | |  | *NA=145* | | | |  | *NA=190* | | | |

***Note:*** *NA is not available*

**Supplementary Material C**

**Table C.1** Association of patient, treatment, and substructure variables and breast toxicity from univariable analysis

| **Variables** | **Breast Toxicity** | | | | | | | | | | | |
| --- | --- | --- | --- | --- | --- | --- | --- | --- | --- | --- | --- | --- |
|  | **Pain** | | | **Oedema** | | | **Atrophy** | | | **Induration** | | |
|  | **Post-RT** | **1 Year** | **2 Year** | **Post-RT** | **1 Year** | **2 Year** | **Post-RT** | **1 Year** | **2 Year** | **Post-RT** | **1 Year** | **2 Year** |
| **Age** | 0.377 | ***0.080*** | ***<0.001*** | 0.176 | 0.794 | 0.189 | ***0.002*** | ***<0.001*** | ***<0.001*** | 0.341 | 0.387 | 0.646 |
| **BMI** | ***0.045*** | 0.967 | 0.477 | ***<0.001*** | ***<0.001*** | ***<0.001*** | 0.881 | ***0.003*** | ***<0.001*** | 0.378 | 0.544 | ***0.080*** |
| **Menopausal status** | 0.634 | 0.687 | 0.219 | ***0.020*** | 0.252 | 0.371 | 0.408 | 0.057 | 0.063 | 0.665 | 0.976 | 0.372 |
| **Smoking** | 0.154 | 0.521 | 0.160 | 0.947 | 0.463 | 0.248 | 0.815 | 0.993 | 0.411 | 0.597 | 0.055 | ***0.047*** |
| **Tumour Size** | 0.382 | 0.384 | 0.388 | 0.291 | ***0.023*** | ***0.004*** | 0.307 | 0.225 | ***0.005*** | 0.438 | 0.131 | ***0.018*** |
| **Breast Volume** | ***0.008*** | 0.459 | 0.484 | ***<0.001*** | ***<0.001*** | ***<0.001*** | 0.332 | 0.165 | ***0.003*** | 0.499 | 0.470 | ***0.001*** |
| **Breast Density** | ***0.011*** | 0.258 | 0.484 | 0.677 | 0.130 | ***0.035*** | 0.098 | ***0.002*** | ***<0.001*** | ***0.001*** | 0.597 | 0.702 |
| **RT Technique** | 0.950 | 0.245 | 0.954 | 0.283 | 0.088 | ***0.036*** | ***<0.001*** | ***0.003*** | 0.050 | ***0.013*** | 0.695 | 0.662 |
| **Fractionation** | 0.846 | 0.123 | ***0.006*** | ***0.003*** | ***0.035*** | 0.423 | ***<0.001*** | ***0.032*** | 0.081 | ***0.002*** | 0.076 | 0.498 |
| **Boost** | 0.502 | 0.504 | 0.123 | ***<0.001*** | ***<0.001*** | 0.126 | ***<0.001*** | ***<0.001*** | 0.220 | ***<0.001*** | ***<0.001*** | ***<0.001*** |
| **Adjuvant Chemotherapy** | 0.279 | 0.822 | 0.133 | 0.514 | ***0.012*** | 0.059 | 0.993 | 0.270 | 0.161 | 0.125 | 0.273 | 0.198 |
| **Fat Volume** | ***0.018*** | 0.472 | 0.431 | ***<0.001*** | ***<0.001*** | ***<0.001*** | 0.275 | 0.115 | ***0.002*** | 0.411 | 0.453 | ***0.003*** |
| **Fibroglandular Volume** | ***0.017*** | 0.608 | 0.868 | ***<0.001*** | ***<0.001*** | ***<0.001*** | 0.830 | 0.785 | 0.139 | 0.965 | 0.735 | ***0.006*** |
| **Mean Dose to Fat** | ***0.027*** | 0.154 | ***0.006*** | ***0.009*** | ***0.013*** | 0.481 | ***<0.001*** | ***0.003*** | 0.377 | ***<0.001*** | ***<0.001*** | ***0.045*** |
| **Mean Dose to Fibroglandular** | 0.341 | 0.268 | ***0.003*** | ***0.012*** | ***0.008*** | 0.406 | ***<0.001*** | ***<0.001*** | 0.630 | ***<0.001*** | ***<0.001*** | ***0.005*** |
| **Mean Dose to Whole Breast** | 0.318 | 0.226 | ***0.003*** | ***0.011*** | ***0.015*** | 0.273 | ***<0.001*** | ***0.002*** | 0.142 | ***<0.001*** | ***<0.001*** | **0.096** |
| **Max Dose to Fat** | 0.518 | 0.583 | ***0.007*** | ***<0.001*** | ***0.003*** | 0.941 | ***<0.001*** | ***<0.001*** | 0.816 | ***<0.001*** | ***<0.001*** | ***<0.001*** |
| **Max Dose to Fibroglandular tissue** | 0.550 | 0.611 | ***0.007*** | ***<0.001*** | ***0.003*** | 0.918 | ***<0.001*** | ***<0.001*** | 0.813 | ***<0.001*** | ***<0.001*** | ***<0.001*** |
| **Max Dose to Whole Breast** | 0.537 | 0.727 | ***0.005*** | ***0.001*** | ***0.004*** | 0.851 | ***<0.001*** | ***<0.001*** | 0.765 | ***<0.001*** | ***<0.001*** | ***<0.001*** |
| **Dose Uniformity of Fat** | 0.832 | 0.970 | 0.081 | ***0.002*** | 0.306 | ***0.048*** | ***<0.001*** | 0.108 | 0.098 | ***<0.001*** | ***0.016*** | 0.686 |
| **Dose Uniformity of Fibroglandular tissue** | 0.328 | 0.991 | 0.425 | ***0.002*** | 0.230 | 0.262 | ***0.003*** | 0.052 | 0.313 | ***<0.001*** | ***0.029*** | 0.396 |
| **Dose Uniformity of Whole Breast** | 0.564 | 0.796 | 0.186 | ***0.004*** | 0.226 | 0.180 | ***<0.001*** | 0.080 | 0.236 | ***<0.001*** | ***0.017*** | 0.661 |

**Aberrations:** BMI is body mass index; RT is radiotherapy; 1 Year is one-year post-RT; 2 Year is two-year post-RT

**Table C2.** The Akaike Information Criteria score for the baseline and the best model from multivariate ordinal logistic regression of each breast toxicity at each timepoint.

| **Toxicity** | **Timepoint** | **AIC Score** | | **∆AIC** |
| --- | --- | --- | --- | --- |
|  |  | **Baseline Model** | **Best Model** |  |
| **Pain** | Post-RT | 1757.5 | 1755.3 | 2.2 |
|  | 1Year-postRT | 1420.3 | - | - |
|  | 2Year-postRT | 1277.0 | - | - |
| **Oedema** | Post-RT | 1244.5 | 1241.8 | 2.7 |
|  | 1Year-postRT | 909.6 | 909.2 | 0.4 |
|  | 2Year-postRT | 619.9 | - | - |
| **Atrophy** | Post-RT | 1354.1 | 1349.8 | 4.3 |
|  | 1Year-postRT | 1353.3 | 1353.2 | 0.1 |
|  | 2Year-postRT | 1586.3 | 1584.7 | 1.6 |
| **Induration** | Post-RT | 1387.3 | 1386.0 | 1.3 |
|  | 1Year-postRT | 1510.4 | 1506.2 | 4.2 |
|  | 2Year-postRT | 1398.9 | 1395.9 | 3.0 |

**Table C.3** shows the multicollinearity of clinical and dosimetric variables as determined by Spearman’s Rank test correlation.

|  | **Age** | **BMI** | **Breast Volume** | **Breast Density** | **Fat Volume** | **Fibroglandular Volume** | **Menopausal Status** | **Smoking** | **Tumour Size** | **Chemotherapy** | **Fractionation** | **Boost Status** | **RT Technique** | **DMean_Breast** | **DMean_Fat** | **DMean_Fibroglandular** | **DMax_Breast** | **DMax_Fat** | **DMax_Fibroglandular** | **DUniformity_Breast** | **DUniformity_Fat** | **DUniformity_Fibroglandular** |
| --- | --- | --- | --- | --- | --- | --- | --- | --- | --- | --- | --- | --- | --- | --- | --- | --- | --- | --- | --- | --- | --- | --- |
| **Age** | 1.00 |  |  |  |  |  |  |  |  |  |  |  |  |  |  |  |  |  |  |  |  |  |
| **BMI** | 0.19 | 1.00 |  |  |  |  |  |  |  |  |  |  |  |  |  |  |  |  |  |  |  |  |
| **Breast Volume** | 0.18 | 0.76 | 1.00 |  |  |  |  |  |  |  |  |  |  |  |  |  |  |  |  |  |  |  |
| **Breast Density** | -0.24 | -0.46 | -0.44 | 1.00 |  |  |  |  |  |  |  |  |  |  |  |  |  |  |  |  |  |  |
| **Fat Volume** | 0.23 | 0.81 | 0.90 | -0.60 | 1.00 |  |  |  |  |  |  |  |  |  |  |  |  |  |  |  |  |  |
| **Fibroglandular Volume** | -0.04 | 0.47 | 0.61 | 0.02 | 0.52 | 1.00 |  |  |  |  |  |  |  |  |  |  |  |  |  |  |  |  |
| **Menopausal Status** | 0.67 | 0.20 | 0.19 | -0.27 | 0.24 | -0.03 | 1.00 |  |  |  |  |  |  |  |  |  |  |  |  |  |  |  |
| **Smoking** | -0.21 | -0.15 | -0.15 | 0.07 | -0.14 | -0.11 | -0.12 | 1.00 |  |  |  |  |  |  |  |  |  |  |  |  |  |  |
| **Tumour Size** | 0.11 | 0.08 | 0.08 | -0.01 | 0.10 | 0.02 | 0.00 | -0.08 | 1.00 |  |  |  |  |  |  |  |  |  |  |  |  |  |
| **Chemotherapy** | -0.12 | -0.04 | -0.08 | 0.00 | -0.05 | -0.13 | -0.10 | 0.04 | 0.29 | 1.00 |  |  |  |  |  |  |  |  |  |  |  |  |
| **Fractionation** | 0.27 | 0.17 | 0.17 | -0.12 | 0.16 | 0.08 | 0.28 | -0.07 | 0.02 | -0.09 | 1.00 |  |  |  |  |  |  |  |  |  |  |  |
| **Boost Status** | -0.17 | -0.19 | -0.27 | 0.14 | -0.17 | -0.14 | -0.20 | 0.10 | 0.19 | 0.17 | -0.45 | 1.00 |  |  |  |  |  |  |  |  |  |  |
| **RT Technique** | -0.05 | 0.14 | 0.22 | -0.11 | 0.19 | 0.10 | 0.06 | 0.00 | 0.14 | 0.01 | 0.34 | -0.10 | 1.00 |  |  |  |  |  |  |  |  |  |
| **DMean_Breast** | -0.24 | -0.16 | -0.17 | 0.10 | -0.17 | -0.08 | -0.25 | 0.09 | 0.02 | 0.09 | -0.85 | 0.64 | -0.34 | 1.00 |  |  |  |  |  |  |  |  |
| **DMean_Fat** | -0.22 | -0.12 | -0.13 | 0.06 | -0.12 | -0.07 | -0.24 | 0.08 | 0.03 | 0.10 | -0.85 | 0.62 | -0.34 | 0.99 | 1.00 |  |  |  |  |  |  |  |
| **DMean_Fibroglandular** | -0.24 | -0.15 | -0.17 | 0.07 | -0.15 | -0.08 | -0.24 | 0.09 | 0.03 | 0.10 | -0.84 | 0.66 | -0.32 | 0.99 | 0.97 | 1.00 |  |  |  |  |  |  |
| **DMax_Breast** | -0.26 | -0.21 | -0.27 | 0.12 | -0.21 | -0.11 | -0.27 | 0.11 | 0.04 | 0.11 | -0.77 | 0.76 | -0.33 | 0.92 | 0.91 | 0.92 | 1.00 |  |  |  |  |  |
| **DMax_Fat** | -0.25 | -0.20 | -0.26 | 0.11 | -0.20 | -0.10 | -0.27 | 0.11 | 0.04 | 0.11 | -0.77 | 0.76 | -0.33 | 0.92 | 0.91 | 0.92 | 1.00 | 1.00 |  |  |  |  |
| **DMax_Fibroglandular** | -0.26 | -0.21 | -0.27 | 0.12 | -0.22 | -0.10 | -0.27 | 0.12 | 0.04 | 0.11 | -0.76 | 0.77 | -0.33 | 0.92 | 0.91 | 0.92 | 1.00 | 0.99 | 1.00 |  |  |  |
| **DUniformity_Breast** | 0.21 | 0.25 | 0.35 | -0.10 | 0.25 | 0.17 | 0.18 | -0.08 | 0.05 | 0.01 | 0.57 | -0.43 | 0.28 | -0.42 | -0.40 | -0.44 | -0.53 | -0.53 | -0.53 | 1.00 |  |  |
| **DUniformity_Fat** | 0.24 | 0.28 | 0.37 | -0.14 | 0.28 | 0.16 | 0.21 | -0.08 | 0.05 | 0.01 | 0.63 | -0.43 | 0.29 | -0.47 | -0.44 | -0.49 | -0.56 | -0.56 | -0.56 | 0.98 | 1.00 |  |
| **DUniformity_Fibroglandular** | 0.17 | 0.23 | 0.32 | -0.08 | 0.22 | 0.17 | 0.14 | -0.08 | 0.05 | 0.03 | 0.50 | -0.41 | 0.26 | -0.37 | -0.36 | -0.37 | -0.48 | -0.48 | -0.48 | 0.96 | 0.92 | 1.00 |

***Note:** BMI is body mass index; RT is radiotherapy; DMean is mean dose; DMax is maximum dose; DUniformity is dose uniformity

**Table C.4** The backward stepwise best-performing model from multivariable ordinal logistic regression showing the association between variables and breast toxicity outcome at each time point for breast pain, breast oedema, breast atrophy, and breast induration.

| **Toxicity** | **Variables** | **Odd ratio (95% CI)** | | |
| --- | --- | --- | --- | --- |
|  |  | **Post-RT** | **1Year post-RT** | **2 Year post-RT** |
| **Breast Pain** | **Age**  **Adjuvant chemotherapy**  **BMI**  **Boost**  **Breast volume**  **Breast density**  **Fractionation**  **Irradiation technique**  **Smoking**  **Tumour size**  **Fibroglandular volume**  **Fat volume**  **Maximum dose to fat**  **Menopausal status**  **Mean dose to fibroglandular**  **Dose uniformity of fat** | 0.982 (0.966-0.998)*  1.263 (0.881-1.810)  1.005 (0.968-0.998)  1.602 (0.995-2.580)  1.000 (0.999-1.000)  0.995 (0.988-1.001)  0.723 (0.442-1.181)  1.048 (0.782-1.402)  1.277 (0.876-1.860)  1.009 (0.996-1.022)  1.001 (0.999-1.003)  1.001 (0.999-1.002)  1.033 (1.001-1.066)*  0.791 (0.516-1.213)  -  - | 0.976 (0.958-0.994)*  1.018 (0.684-1.515)  0.983 (0.941-1.027)  0.949 (0.949-1.867)  1.000 (0.999-1.000)  0.996 (0.989-1.003)  1.363 (0.915-2.029)  1.232 (0.899-1.690)  0.902 (0.594-1.371)  1.011 (0.997-1.025)  1.001 (0.999-1.003)  1.001 (0.999-1.002)  -  0.717 (0.448-1.147)  -  1.006 (0.995-1.017) | 0.971 (0.951-0.991)*  0.885 (0.591-1.325)  1.003 (0.956-1.053)  1.242 (0.797-1.934)  1.000 (0.999-1.000)  0.999 (0.992-1.006)  1.030 (0.552-1.923)  0.976 (0.691-1.378)  1.015 (0.654-1.574)  1.009 (0.995-1.024)  1.000 (0997-1.002)  1.001 (0.999-1.002)  -  0.777 (0.471-1.282)  1.031 (0.983-1.082)  - |

**Note:** * *p<*0.05, ** *p<*0.01

**Table C.4** The backward stepwise best-performing model from multivariable ordinal logistic regression showing the association between interested variables and breast toxicity outcome at each time point for breast pain, breast oedema, breast atrophy, and breast induration. (cont.)

| **Toxicity** | **Variables** | **Odd ratio (95% CI)** | | |
| --- | --- | --- | --- | --- |
|  |  | **Post-RT** | **1Year post-RT** | **2 Year post-RT** |
| **Breast Oedema** | **Age**  **Adjuvant chemotherapy**  **BMI**  **Boost**  **Breast volume**  **Breast density**  **Fractionation**  **Irradiation technique**  **Smoking**  **Tumour size**  **Fibroglandular volume**  **Fat volume**  **Menopausal status**  **Mean dose to breast**  **Dose uniformity of fat** | 1.007 (0.988-1.026)  1.202 (0.795-1.816)  1.004 (0.964-1.046)  0.708 (0.542-1.081)  0.999 (0.998-0.999)*  1.006 (1.000-1.012)*  1.257 (0.839-1.883)  0.708 (0.510-0.982)**  0.883 (0.583-1.337)  1.004 (0.989-1.018)  1.005 (1.002-1.007)**  1.003 (1.002-1.005)**  1.458 (0.903-2.354)  -  0.988 (0.976-0.999)* | 1.025 (1.001-1.051)*  0.627 (0.393-1.000)  0.973 (0.924-1.025)  0.448 (0.285-0.705)*  1.001 (1.000-1.001)  1.002 (0.993-1.011)  1.710 (1.035-2.825)*  0.793 (0.533-1.180)  0.722 (0.430-1.212)  1.001 (0.983-1.018)  1.002 (1.000-1.005)  1.001 (1.000-1.003)*  1.759 (0.963-3.211)  -  1.009 (0.995-1.024) | 1.007 (0.977-1.037)  0.630 (0.354-1.121)  1.013 (0.977-1.037)  0.413 (0.233-0.734)*  1.001 (1.000-1.002)*  1.003 (0.991-1.016)  0.904 (0.551-1.485)  0.904 (0.551-1.485)  1.142 (0.546-2.388)  1.008 (0.987-1.029)  1.003 (1.001-1.006)*  1.001 (0.999-1.003)  0.630 (0.354-1.121)  0.979 (0.921-1.042)  - |

**Note:** * *p<*0.05, ** *p<*0.01

**Table C.4** The backward stepwise best-performing model from multivariable ordinal logistic regression showing the association between interested variables and breast toxicity outcome at each time point for breast pain, breast oedema, breast atrophy, and breast induration. (cont.)

| **Toxicity** | **Variables** | **Odd ratio (95% CI)** | | |
| --- | --- | --- | --- | --- |
|  |  | **Post-RT** | **1Year post-RT** | **2 Year post-RT** |
| **Breast Atrophy** | **Age**  **Adjuvant chemotherapy**  **BMI**  **Boost**  **Breast volume**  **Breast density**  **Fractionation**  **Irradiation technique**  **Smoking**  **Tumour size**  **Fibroglandular volume**  **Fat volume**  **Maximum dose to fibroglandular**  **Menopausal status**  **Mean dose to fibroglandular** | 1.037 (1.017-1.058)**  1.251 (0.825-1.897)  1.072 (1.026-1.119)*  0.752 (0.492-1.149)  0.997 (0.996-0.998)**  0.988 (0.980-0.995)*  0.975 (0.534-1.780)  1.937 (1.381-2.717)**  0.898 (0.582-1.387)  1.012 (0.997-1.028)  1.005 (1.002-1.007)**  1.002 (1.001-1.004)*  -  1.326 (0.797-2.207)  1.057 (1.011-1.105)* | 1.037 (1.017-1.057)**  0.965 (0.641-1.454)  1.098 (1.049-1.149)**  0.644 (0.425-0.976)*  0.997 (0.997-0.998)**  0.989 (0.981-0.997)**  0.940 (0.521-1.697)  1.225 (0.879-1.709)  0.843 (0.544-1.306)  1.007 (0.992-1.022)  1.004 (1.002-1.006)**  1.002 (1.001-1.003)**  -  1.267 (0.764-2.100)  1.030 (0.987-1.076) | 1.030 (1.011-1.049)**  0.839 (0.578-1.219)  1.084 (1.038-1.132)**  1.157 (0.692-1.936)  0.998 (0.997-0.999)**  0.993 (0.986-0.999)*  0.583 (0.350-0.971)*  0.722 (0.529-0.984)*  1.091 (0.718-1.658)  1.008 (0.994-1.022)  1.003 (1.001-1.005)**  1.002 (1.000-1.003)**  1.032 (0.998-1.066)  1.385 (0.869-2.207)  - |

***Note:*** * *p<*0.05, ** *p<*0.01

**Table C.4** The backward stepwise best-performing model from multivariable ordinal logistic regression showing the association between interested variables and breast toxicity outcome at each time point for breast pain, breast oedema, breast atrophy, and breast induration. (cont.)

| **Toxicity** | **Variables** | **Odd ratio (95% CI)** | | |
| --- | --- | --- | --- | --- |
|  |  | **Post-RT** | **1Year post-RT** | **2 Year post-RT** |
| **Breast Induration** | **Age**  **Adjuvant chemotherapy**  **BMI**  **Boost**  **Breast volume**  **Breast density**  **Fractionation**  **Irradiation technique**  **Smoking**  **Tumour size**  **Fibroglandular volume**  **Fat volume**  **Menopausal status**  **Mean dose to fibroglandular**  **Dose uniformity of fibroglandular** | 1.002 (1.004-1.041)*  1.681 (1.116-2.531)*  1.030 (0.988-1.073)  0.407 (0.290-0.571)**  0.998 (0.997-0.999)**  1.008 (1.001-1.014)*  1.031 (0.723-1.472)  1.024 (0.749-1.400)  0.932 (0.621-1.398)  0.995 (0.980-1.010)  1.003 (1.001-1.005)**  1.003 (1.001-1.004)**  1.035 (0.642-1.668)  -  0.992 (0.983-1.001) | 1.019 (1.001-1.038)*  1.046 (0.713-1.535)  1.031 (0.989-1.075)  0.520 (0.355-0.761)**  0.999 (0.998-0.999)**  1.003 (0.997-1.010)  0.616 (0.358-1.059)  0.763 (0.559-1.042)  0.681 (0.457-1.016)  1.004 (0.989-1.018)  1.002 (1.000-1.004)*  1.002 (1.000-1.003)*  1.060 (0.662-1.698)  1.051 (1.010-1.093)*  - | 1.000 (0.981-1.019)  0.974 (0.661-1.436)  1.000 (0.957-1.046)  0.389 (0.272-0.557)**  0.999 (0.998-0.999)**  1.005 (0.998-1.011)  0.820 (0.569-1.183)  1.034 (0.753-1.421)  0.704 (0.464-1.069)  1.007 (0.992-1.022)  1.003 (1.001-1.005)**  1.003 (1.002-1.004)**  0.714 (0.441-1.157)  -  1.011 (1.001-1.021)* |

***Note:*** * *p<*0.05, ** *p<*0.01


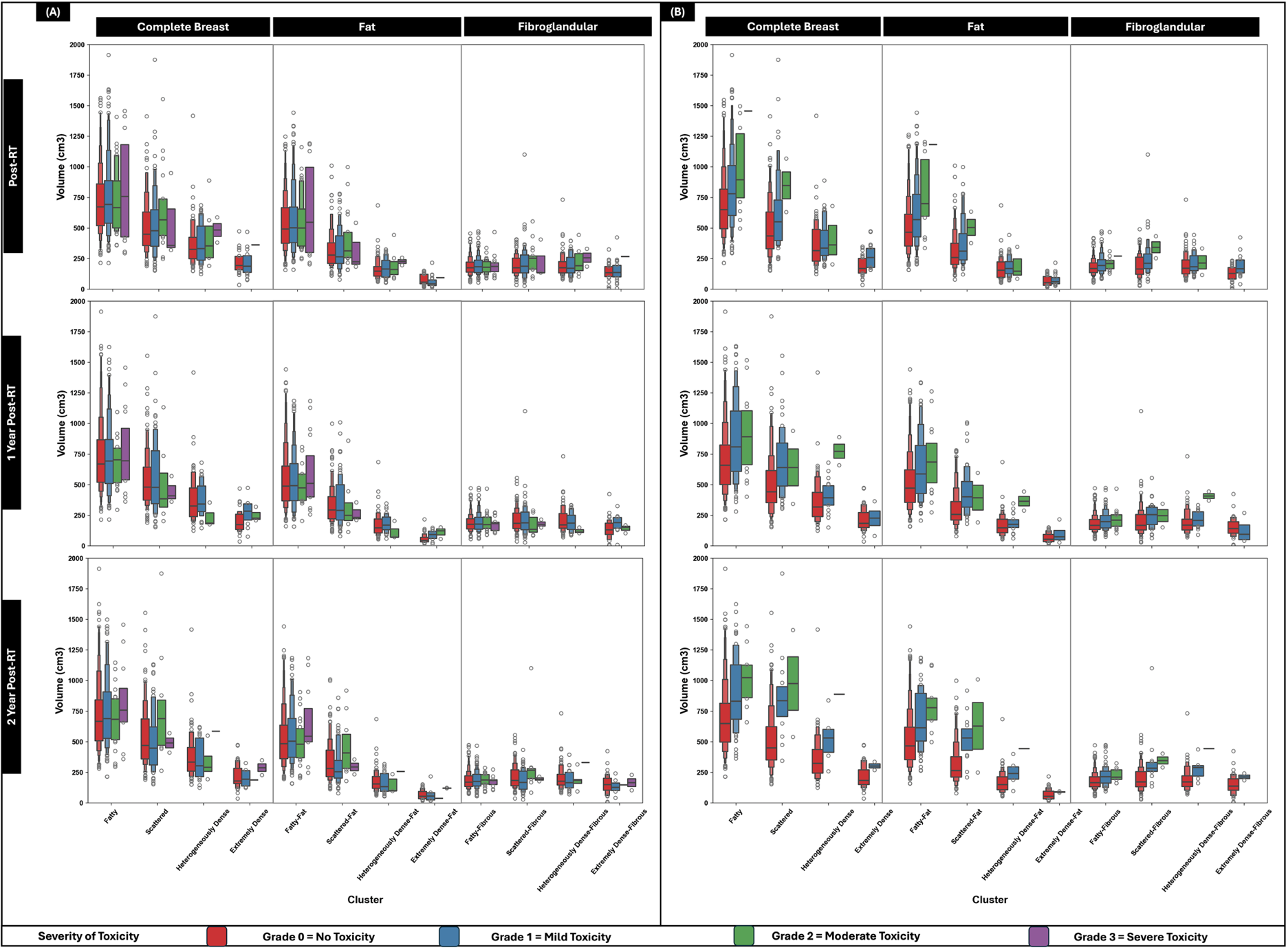


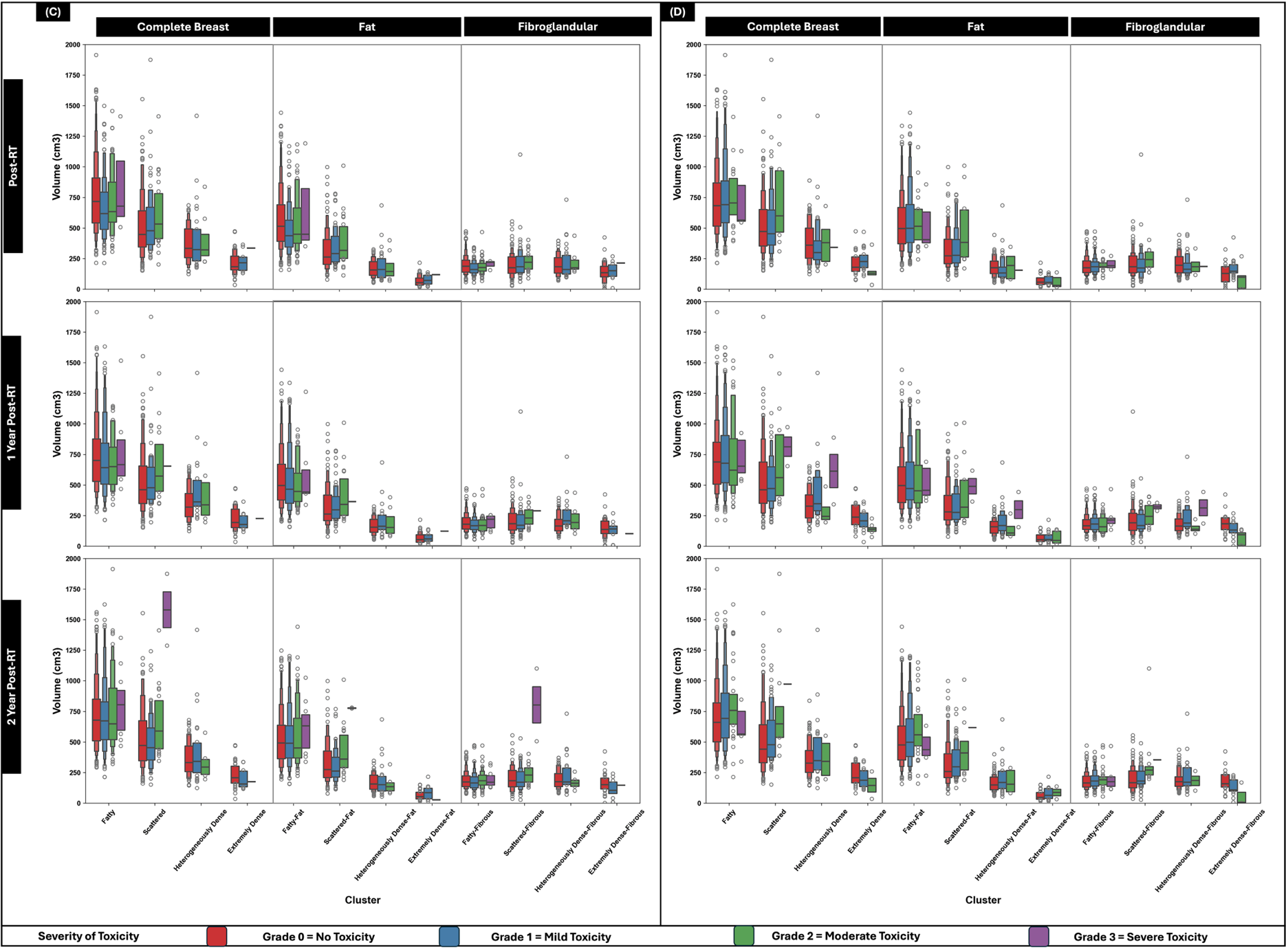


**Figure C.1** Volume of the complete breast, fat, and fibroglandular tissue from each breast density cluster in each toxicity severity level of (A) breast pain, (B) breast oedema, (C) breast atrophy, and (D) breast induration.


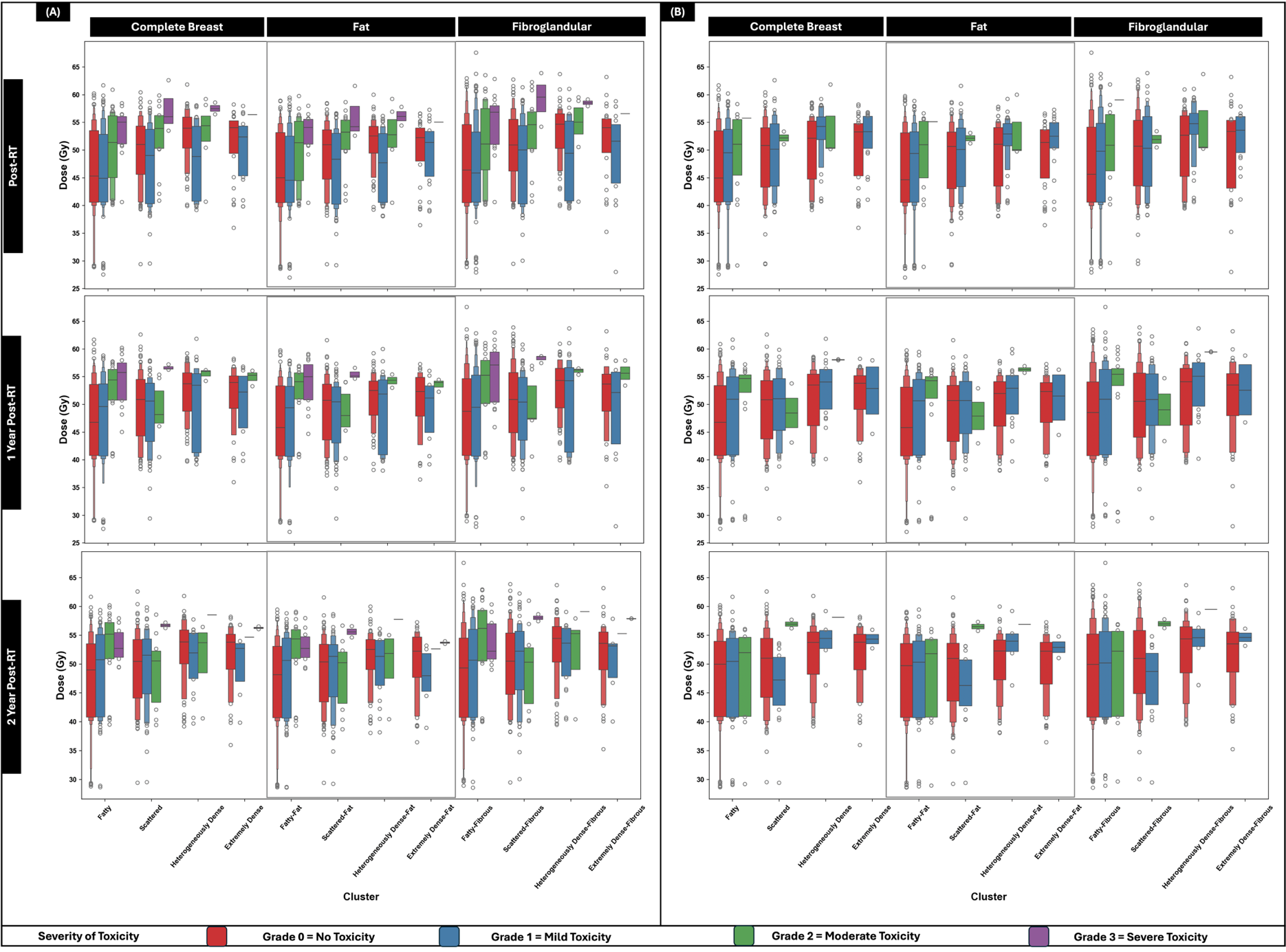


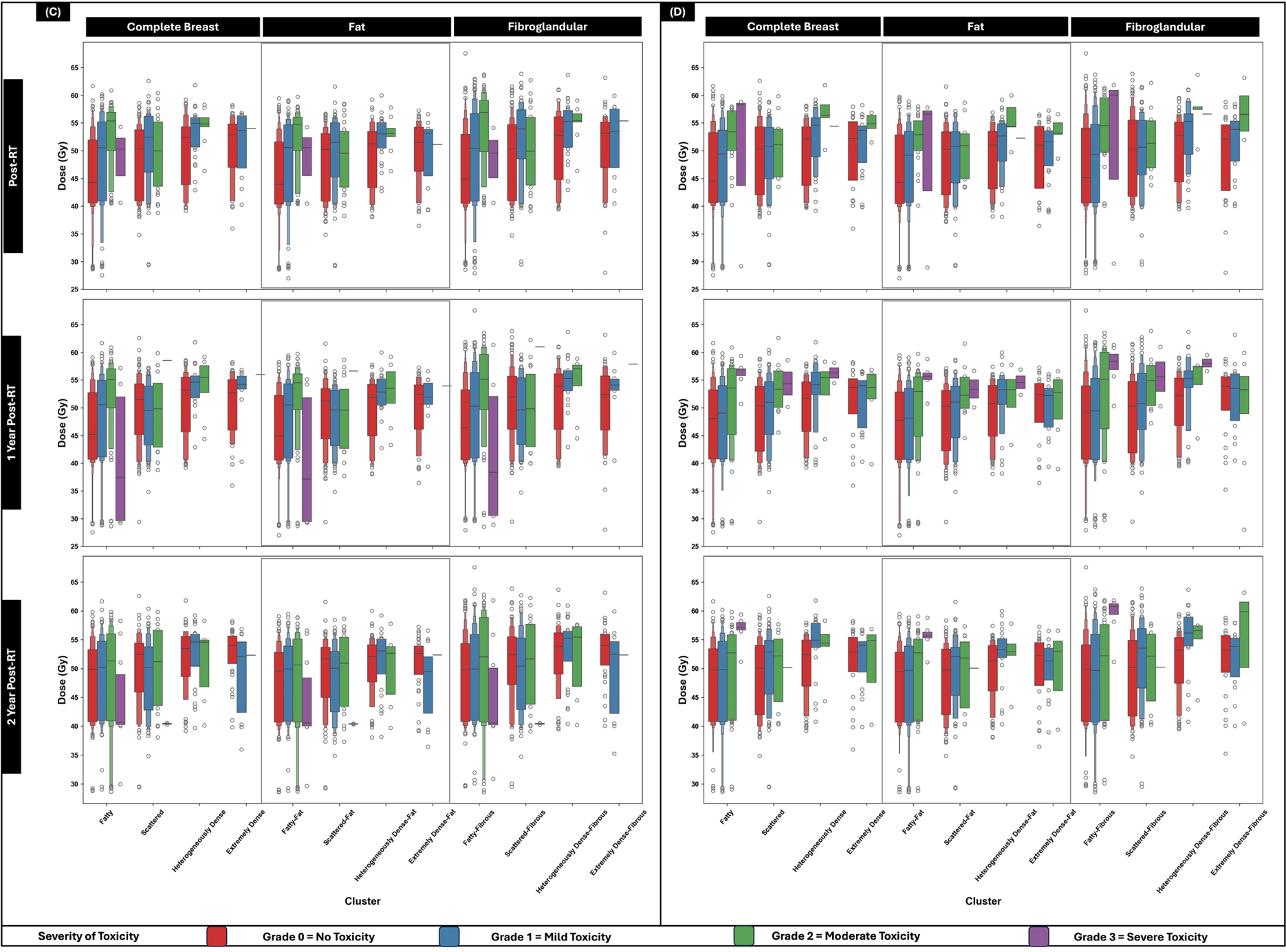


**Figure C.2** Mean dose deposit to the complete breast, fat, and fibroglandular tissue from each breast density cluster in each toxicity severity level of (A) breast pain, (B) breast oedema, (C) breast atrophy, and (D) breast induration.


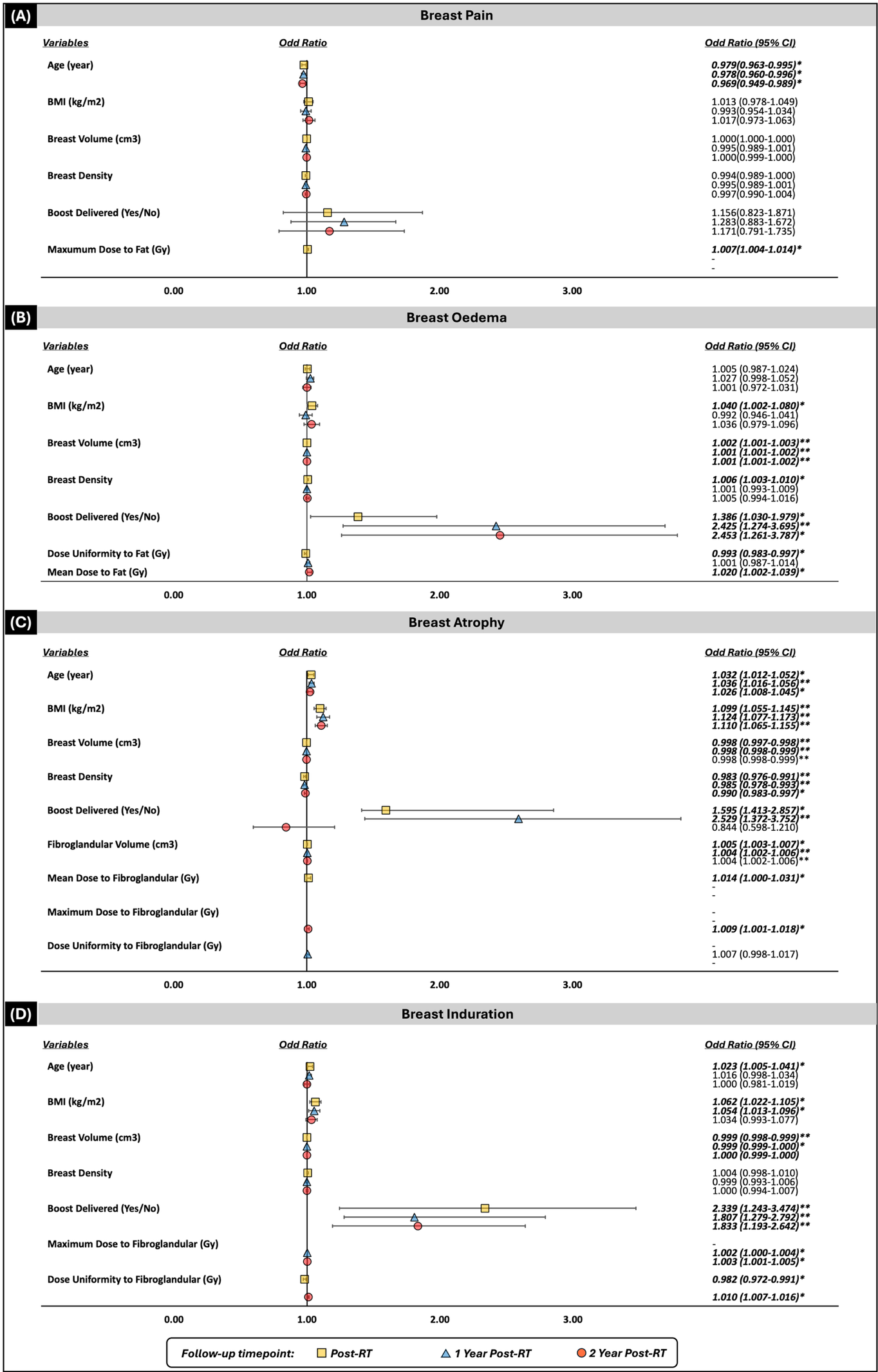


**Figure C.3** The best-performing models from multivariable ordinal logistic regression based on EQD_2_ showing the association between studied variables and breast toxicity outcomes at each time point for (A) pain, (B) oedema, (C) atrophy, and (D) induration.

Note: **p<0.05* and ***p<0.001*
